# The neuroprotective mechanisms of ginkgolides and bilobalide in cerebral ischemic injury: a literature review

**DOI:** 10.1186/s10020-019-0125-y

**Published:** 2019-12-21

**Authors:** Zili Feng, Qian Sun, Wang Chen, Yu Bai, Daihua Hu, Xin Xie

**Affiliations:** 1 School of Bioscience and Engineering, Shaanxi University of Technology, No.1 Donghuan 1st Road, Hanzhong, 732001 People’s Republic of China; 2 Key Laboratory of Resource Biology and Biotechnology in Western China, Ministry of Education, College of Life Science, Northwest University, Xi’an, 710069 People’s Republic of China

**Keywords:** Ischemic stroke, Ginkgolides, Bilobalide, Neuroprotective effect, Cerebral injury

## Abstract

The incidence and mortality of strokes have increased over the past three decades in China. Ischemic strokes can cause a sequence of detrimental events in patients, including increased permeability and dysfunction of the blood-brain barrier, brain edema, metabolic disturbance, endoplasmic reticulum stress, autophagy, oxidative stress, inflammation, neuron death and apoptosis, and cognitive impairment. Thrombolysis using recombinant tissue plasminogen activator (rtPA) and mechanical embolectomy with a retrievable stent are two recognized strategies to achieve reperfusion after a stroke. Nevertheless, rtPA has a narrow therapeutic timeframe, and mechanical embolectomy has limited rates of good neurological outcomes. EGb761 is a standardized and extensively studied extract of *Ginkgo biloba* leaves. The ginkgolides and bilobalide that constitute a critical part of EGb761 have demonstrated protective properties towards cerebral injury. Ginkgolides include Ginkgolide A (GA), Ginkgolide B (GB), Ginkgolide C (GC), Ginkgolide J (GJ), Ginkgolide K (GK), Ginkgolide L (GL), and Ginkgolide M (GM). This review seeks to elucidate the neuroprotective effects and mechanisms of ginkgolides, especially GA and GB, and bilobalide in cerebral injury following ischemic strokes.

## Background

In this review, we hope to provide the readers with an overview of current understanding of the neuroprotective effects and mechanisms of ginkgolides and bilobalide in cerebral injury following ischemic strokes. Ginkgolides and bilobalide are unique terpenoid components of the *Ginkgo biloba* tree. Ginkgolides include Ginkgolide A (GA), Ginkgolide B (GB), Ginkgolide C (GC), Ginkgolide J (GJ), Ginkgolide K (GK), Ginkgolide L (GL), and Ginkgolide M (GM). Extensive evidences have shown that *Ginkgo biloba* extracts have neuroprotective properties under conditions such as hypoxia/ischemia, seizure activity and peripheral nerve damage. Although its various neurobiological effects, their macromolecular targets in brain require further study. Knowledgement of binding targets would help to understand the mechanism by which *Ginkgo biloba* extracts modulate neurol and congnitive functions. Using photoaffinity-labeling, Akira Kawamura et al. found that ginkgolides are a new class of microtubule-modulating agents with distinct effects on α-tubulin biology (Kawamura et al. 2017). Research on the biochemical effects and mechanism of *Ginkgo biloba* extracts is still at a very early stage. However, all the published articles suggest that *Ginkgo biloba* extracts are worthy of further investigation as potential neuroprotectants.

## Cerebral ischemic injury and treatment

It is well-known that the human brain consumes a large part of the energy produced by the body. However, the brain is unable to store sufficient energy to meet its needs and is vulnerable to interruptions in the oxygen and glucose supplies. Cerebral ischemia causes irreversible neurological damage resulting in permanent disabilities. The incidence and mortality of cerebral ischemia have increased over the past three decades in China. In a study that surveyed 480,687 adults in 31 provinces in China, the annual rate of age-standardized prevalence, incidence, and mortality rates were 1114.8, 246.8, 114.8/100000, respectively. Ischemic strokes accounted for 69.6 and 77.8% of the incident and prevalent strokes, respectively (Wang et al. 2017). Although much attention has been paid to treating ischemic strokes, the disease remains a heavy burden on patients and the society.

Currently, recanalization and reperfusion strategies are considered the most effective interventions for acute ischemic strokes. Thrombolysis using recombinant tissue plasminogen activator (rtPA) and mechanical embolectomy with a retrievable stent are two widely recognized strategies for achieving reperfusion (Rabinstein 2017). rtPA can degrade fibrin clots by catalyzing the conversion of plasminogen to plasmin. However, rtPA has a narrow therapeutic timeframe of 3 to 4.5 h, and the clinical use of rtPA for acute ischemic stroke has been constrained to a relatively small patient population (less than 5%) (Sifat et al. 2017). Adverse effects of rtPA treatment have also been reported. For acute ischemic stroke, the most feared complication is symptomatic intracerebral hemorrhage (Yaghi et al. 2014). Induction of NLRP3 inflammasome in neurons, microglia and endothelial cells, degradation of BBB components, and hemorrhagic transformation were reported after delayed rtPA treatment in rats with thromboembolic focal cerebral ischemia (Guo et al. 2018). Necrotic changes in neurons with a disruption of the microvascular basal lamina and increased edema formation were also demonstrated after rtPA injection in rats (Goto et al. 2007). While, mechanical thrombectomy combined with rtPA has proven its effectiveness in the therapeutic management of acute ischemic strokes. Despite the breakthroughs leading to higher recanalization rates and acceptable safety, there is still quite a bit of room for improvements, as accessibility to mechanical thrombectomy remains limited (Derex and Cho 2017).

## Composition of *Ginkgo biloba* extract

**Fig. 1:**
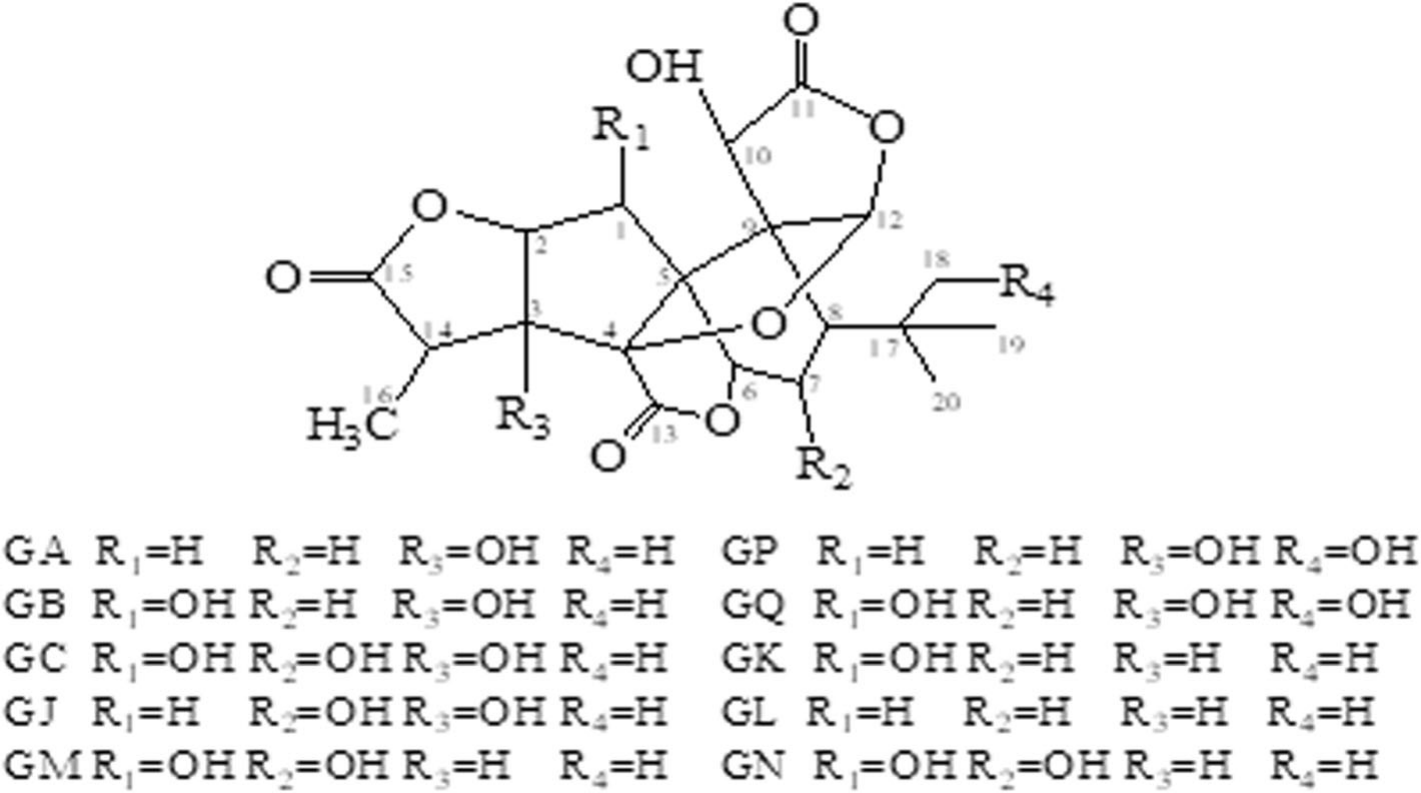
Structures of ginkgolides

*Ginkgo biloba* is an ancient Chinese tree that has long been used for the therapy of multiple diseases. The standardized Ginkgo extract, EGb761, is a well-known extract of *G. biloba* leaves dating back a century. Studies have been shown its neuroprotective effects against various cardiovascular and neurological disorders, such as ischemia, Alzheimer’s disease and depression. EGb-761 contains two groups of bioactive constituents: 24% of flavonol glycosides and 6% of terpene trilactones. Ginkgolides and bilobalide are major constituents of the terpene trilactones (Jaracz et al. 2004). For years, researchers have prepared ginkgolides, which include Ginkgolide A (GA), Ginkgolide B (GB), Ginkgolide C (GC), Ginkgolide J (GJ), Ginkgolide K (GK), Ginkgolide L (GL), and Ginkgolide M (GM), and investigated their effectiveness towards multiple diseases (Fig. 1). Bilobalide is a sesquiterpene and an active constituent of *G. biloba* extract. This review focuses on the effects and mechanisms of ginkgolides and bilobalide in the treatment of cerebral ischemia.

## Mechanisms of ginkgolides and bilobalide in neuron protection after cerebral ischemia

### Increased permeability and dysfunction of the blood-brain barrier

The blood brain barrier (BBB) helps to maintain a suitable environment for the central nervous system and prevents exposure to toxins and pathogens. Cerebral ischemia results in a breakdown of the BBB, compromising the integrity of the BBB. In this process, the constituents of the BBB, including endothelial cells with tight junction proteins, astrocytes, pericytes, and the basement membrane, are negatively affected (Haley and Lawrence 2017). The disruption of the BBB is followed by more serious clinical consequences like hemorrhagic transformation in the ischemic cerebral tissues (Kassner and Merali 2015). People have developed many methods to assess BBB disruption after ischemic strokes, including the assessment of cell morphology, protein expression and localization, cell electrophysiology, and gross neurological function (Kassner and Merali 2015).

The therapeutic neuroprotection effects of *G. biloba* extract and GB on BBB function are being investigated in vitro and in vivo to evaluated its clinical use. In endothelial cells, *G. biloba* extract and GB solution reduced the endothelial permeability coefficients and upregulated the expression of tight junction proteins such as ZO-1 and occludin (Fang et al. 2010). In rats with middle cerebral artery occlusion (MCAO), i.v. administration via vena caudalis demonstrated higher concentration of brain GB in MCAO rats than that of normal rats, indicating that BBB permeability for GB was increased after ischemia-reperfusion injury (Fang et al. 2010). Another research reported that intraperitoneal injection of GB in MCAO rats could decrease the neurological deficit score, increase the proportion of nestin-, neuron-specific enolase- and glial fibrillary acid protein-positive cells, increase the mRNA expression of brain-derived neurotrophic factor and epidermal growth factor, and increase the expression levels of brain-derived neurotrophic factor and suppressor of cytokine signaling 2 in the ischemic penumbra (Zheng et al. 2018a). GB cinnamate, a synthesized GB analog, had increased BBB permeation compared to GB, with a 1.61-fold increase in half-life of Sprague Dawley (SD) rats (Lu et al. 2015). 10-O-(N, N-dimethylaminoethyl)-ginkgolide B methanesulfonate, also called XQ-1H, is a derivate of GB. Pre-administration with XQ-1H suppressed hyperlipidemia, reduced cerebral infarct size, improved BBB permeability and diminished brain edema after a stroke in hyperlipidemic rats (Fang et al. 2015). In rats with hyperthermic brain injury, pretreatment with EGB-761 or its constituent, GB, significantly reduced the BBB permeability, brain edema and cell injury (Sharma et al. 2000).

### Brain edema

Experiments conducted on humans and animals have demonstrated that oxygen and glucose deprivation, reperfusion or reoxygenation (OGD/R) injury, and vasopressin (VP) hypersecretion are primary causes of brain edema formation after ischemic strokes (O'donnell et al. 2013). A luminal BBB Na-K-Cl cotransporter (NKCC) and Na/H exchanger (NHE) are reported to be critical for edema formation during cerebral ischemia. The NKCC and NHE are expressed in the BBB membrane and can be induced by hypoxia, aglycemia, and arginine vasopressin. The treatment by inhibitors for NKCC and NHE can lead to edema reduction and injury alleviation (O’donnell et al. 2004, 2013). Furthermore, aquaporin 4 is a membrane water channel that is largely expressed in glial cells and near cerebral capillaries and pial membranes. The role of aquaporin 4 in brain edema formation during stroke has been reviewed (Zador et al. 2009).

Oral administration of *G. biloba* extract (Ph-Gb) effectively reduced water content in the hippocampus of gerbils in a dose-dependent manner (Calapai et al. 2000). GB, whether administered before or after stroke, will still exert its effect on brain edema. Administration of GB 2 h after reperfusion effectively decreased infarction volume and brain edema in rats with MCAO (Fang et al. 2010). Botao et al. (2013) found that GB pretreatment reduced brain water content, increased superoxide dismutase activity and glutathione concentration, decreased MDA concentration, and decreased active caspase-3 and PARP expression in rats with high altitude cerebral edemas. GK, a derivate of GB, given at a higher dose also attenuated cerebral infarction and repressed brain edema formation in MCAO rats (Ma et al. 2012). In a cerebral ischemia-reperfusion injury (CIRI) model, pretreatment with Ginkgolides at 2.5 ml/kg reduced cerebral infarction area and cerebral edema, partly through TWEAK–Fn14 cytokine receptor axis (Xiao et al. 2019). The effect of bilobalide on brain edemas has been demonstrated in animal experiments. Bilobalide reduced water content and inhibited edema formation in either rat hippocampal slices exposed to oxygen-glucose deprivation or mice brain tissue with MCAO (Mdzinarishvili et al. 2007). The mechanisms by which ginkgolides and bilobalide reduce brain edema need more investigation.

### Brain metabolic disturbance

The infusion of GB in patients with non-traumatic severe acute haemorrhagic stroke can reduce intracranial pressure, augment cerebral perfusion pressure, and decrease lactate/pyruvate ratio (Chi et al. 2015). In tree shrews, GB treatment inhibited neuronal mitochondria cristae disorganization and helped recover mitochondrial respiration after cerebral ischemia (Li et al. 2007). Mitochondria from ischemic brain tissue exhibit an inhibition in respiration and an opening of the mitochondrial permeability transition pore (mPTP). GK attenuated mitochondrial dysfunction, reduced mitochondrial fission, and inhibited mPTP opening through a GSK-3β dependent mechanism in both OGD/R neuroblastoma cells and MCAO mice (Zhou et al. 2017). Pretreatment with bilobalide, before transient MCAO treatment, significantly attenuated the effect (Schwarzkopf et al. 2013a). The activity of mitochondrial complex I from mice brain tissue was also reduced in ischemic conditions but improved by bilobalide treatment (Schwarzkopf et al. 2013a). Although bilobalide showed no effect on the activity of the mitochondrial respiratory chain in aged mice after ischemia, it reduced mitochondrial sensitivity to calcium-induced swelling (Schwarzkopf et al. 2013b). Bilobalide treatment also prevented a decrease in mitochondrial adenosine triphosphate content, cytochrome c oxidase subunit I mRNA, and protein levels and COX activity in hippocampal tissues of ovariectomized rats (Shi et al. 2011). These results also suggested a protective role of bilobalide for neurodegenerative diseases (Shi et al. 2011). MCAO decreased extracellular glucose in the striatum and increased glutamate levels in striatal and hippocampal tissues in a mouse model. Bilobalide did not affect glucose levels but diminished glutamate release in both core and penumbra regions (Lang et al. 2011). Notably, rtPA administration may cause metabolic disturbances in the prefrontal cortex, resulting in increased production of glutamic acid, aspartic acid, N-acetyl-l-aspartic acid, gamma-aminobutyric acid, and glutamine but decreased production of glycine. In contrast, administration of *G. biloba* extract and diterpene ginkgolide effectively ameliorated metabolic disturbances induced by rtPA (Chen et al. 2018a).

### Endoplasmic reticulum stress

Cerebral ischemia perturbs endoplasmic reticulum (ER) function, resulting in accumulation of unfolded proteins in the ER lumen, a condition referred to as ER stress. ER stress is an essential signaling event in the progression of brain ischemia/reperfusion (I/R) injury. Drugs modulating ER stress have been proven to exert a remarkable protective effect on the ischemic brain and offer the prospect of new stroke therapies (Xin et al. 2014). In a rat model of global ischemia, ER stress in the hippocampus was altered both spatially and temporally. In vitro, inducing ER stress increased neuronal death but inhibition of ER stress also produced neurotoxicity. Thus, further studies are needed to address when ER stress becomes detrimental to neurons, and whether modulation of ER stress pharmacologically is a viable therapeutic strategy for cerebral ischemia (Hadley et al. 2018). Thromboembolic stroke can cause PERK/eIF2α signaling activation in the infarct cortical region of a mouse model, and can be suppressed by thrombolysis. However, rtPA can bind to cell surface Grp78 and decrease the PERK/eIF2α activation induced by a stroke (Louessard et al. 2017). Injection of granulocyte-colony stimulating factor into rats with MCAO attenuated the expression of ATF4, ATF6, p-p38MAPK, pJNK, and CHOP, all of which were involved in the ER stress-related apoptosis pathway (Menzie-Suderam et al. 2018).

Alleviation of ER stress by ginkgolides and bilobalide has been demonstrated in animal and human experiments. *G. biloba* extract inhibited cardiomyocyte apoptosis and inflammation in high-fat diet ApoE−/− mice via constraining ER stress related apoptosis pathway, as evidenced by decreasing phosphorylated c-Jun N-terminal kinase (p-JNK), C/EBP homologous protein (CHOP), caspase-12, and cleaved caspase-3 (Tian et al. 2018). In a rat model with myocardial I/R, GB treatment inhibited the expression of p-PERK, p-IRE1α, and ATF6 protein, decreasing the number of apoptotic myocardial cells, while at the same time, it restored p-Akt and p-mTOR expression in these cells, promoting cell survival (Guo et al. 2019). Bilobalide increased the expression of catalase and glutathione and reduced ER stress in human melanocytes treated with H_2_O_2_ (Lu et al. 2016). The effect of ginkgolides on ER stress in neuron cells has not been fully explored. However, in cultured SH-SY5Y cell, preincubation with GB could attenuate bupivacaine-induced mitochondrial dysfunction, ER stress and cell apoptosis (Li et al. 2013).

### Autophagy

Autophagy occurs in response to nutrient starvation or metabolic stress and helps to maintain tissue homeostasis by recycling cytoplasmic proteins and organelles. mTOR complex 1 (mTORC1) and AMPK are essential regulators of autophagy activation. Under the condition of sufficient nutrition, the activity of mTORC1 kinase is vigorous, while the activity of AMPK is depressed. Therefore, ULK1 and Atg13 are phosphorylated and inactivated by mTORC1. In contrast, in a nutrition shortage, AMPK is activated and mTORC1 is suppressed, followed by the activation of ULK1 and Atg13. ULK1 and Atg13 further promote the progression of autophagy (Wang et al. 2018a). Following this, cytoplasmic components are packaged into autophagosomes and then fused with lysosomes, leading to the formation of autolysosomes. Excessive autophagy can be detrimental to neuron survival by promoting apoptosis, while moderate autophagy ameliorates neuronal damage and suppresses apoptosis induced by ischemia (Song et al. 2017). These two effects of autophagy have been shown in animal studies. For example, I/R induced ER stress-dependent autophagy through PERK and IRE1 signaling, and thus neuronal apoptosis in MCAO mice (Feng et al. 2017). In contrast, preconditioning with cortical spreading depression induced AMPK-dependent autophagy and decreased neurological deficits and neuronal apoptosis in MCAO rats (Shen et al. 2017).

Aβ and phosphorylated Tau are two major pathogenic molecules of Alzheimer’s disease (AD). Oligomeric Aβ induces hyperphosphorylation and aggregation of tau and drives tau pathology expanding from a restricted region around medial temporal cortex to the whole neocortex. Using tau-transgenic AD mice, Qin et al. found that treatment with *G. biloba* extract EGb 761 decreased p-Tau level in the brain and shifted microglial activation from pro-inflammatory to anti-inflammatory. The components of GA, bilobalide, and flavonoids, but not GB or GC in EGb 761 can increase LC3B-II protein expression and promote p-Tau degradation in the lysosomes of neurons in these tau-transgenic AD mice (Qin et al. 2018). However, inhibition of ATG5 activity or treatment with Bafilomycin B1 dampened the p-Tau degradation induced by EGb 761 (Qin et al. 2018). In TgCRND8 transgenic AD mice that overexpress Alzheimer’s amyloid precursor protein in neurons, EGb 761 treatment decreased both Aβ-induced microglial secretion of TNF-α and IL-1β and activation of caspase-1 by activating the autophagy process. Inhibition of autophagy in microglia suppressed the effects of EGb 761 (Liu et al. 2015). In an I/R injured rat model, intraperitoneal delivery of bilobalide reduced infarct size, cell apoptosis and autophagy, improved neurological scores, and promoted angiogenesis in the brain (Zheng et al. 2018b). However, there is no direct evidence showing the effect of ginkgolides on neuronal autophagy with I/R damage.

### Oxidative stress

Oxidative stress plays an essential role in the pathogenesis of cerebral ischemia-reperfusion injury, mainly through increased reactive oxygen/nitrogen species (ROS/RNS) production and decreased activity levels of scavenger enzymes and protective antioxidants. During reperfusion, replenished oxygen is crucial for sustaining neuronal viability. However, oxygen is also a substrate for the production of oxygen free radicals (oxidants). Antioxidant defenses systems, including superoxide dismutase (SOD) and glutathione (GSH), process the scavenging of oxidants, and alleviate oxidant-mediated brain damage. When reactive ROS/RNS surpasses the scavenging capacity of antioxidant defenses systems, oxidative stress takes place with adverse effects on neuron function and survival. Nakano et al. (2017) observed that cerebral ROS generation peaked 1 day after transient MCAO in mice, coinciding with an increase in Nrf2, a transcription factor that regulated antioxidant enzymes. Oxidative stress leads directly to DNA damage that occurs within minutes after cerebral ischemic strokes. On one hand, DNA damage triggers pro-death signaling pathways and results in apoptosis or necrosis, impairing recovery of neuron function. While on the other hand, oxidative DNA damage also activates the DNA repair system that is critical for neurogenesis, angiogenesis, axonal outgrowth, and remyelination (Li et al. 2018).

In both undifferentiated and differentiated SH-SY5Y neuronal cells, pretreatment of GB could counter Aβ1–42-induced oxidative stress responses through hampering production of ROS and RNS, restoring antioxidant activities of SOD and GSH, and maintaining genome integrity by reducing the oxidative DNA and RNA base damages (Gill et al. 2017). In exogenous H_2_O_2_-treated PC12 cells, pretreatment with GK notably attenuated ROS level (Ma et al. 2012). In MCAO rat, GK increased SOD activity, decreased MDA content and reduced NO content and NOS activity in the serum and ischemic brain sections (Ma et al. 2012). In MCAO rats, GB treatment could significantly increase the expressions of anti-oxidative stress-related proteins, including HO-1, Nqo1 and SOD, as well as Nrf2, the transcription factor that regulates antioxidant enzymes (Liu et al. 2019). GB but not bilobalide restored cerebral blood flow and reduced reactive oxygen species in hyperglycemic rats (Huang et al. 2012). Diterpene ginkgolides (GA, GB and GC) activated Akt signaling and led to the nuclear location of Nrf2 and phosphorylation of CREB, which had protective effects against I/R injury (Zhang et al. 2018).

### Inflammation

Ischemic strokes can interrupt the balance between the pro-inflammatory and anti-inflammatory responses in the brain. Bone marrow derived cells like neutrophils, monocytes/macrophages, and lymphocytes can infiltrate the ischemic brain and release a large number of pro-inflammatory factors, including inducible nitric oxide synthase (iNOS), matrix metalloproteinases (MMPs), pro-inflammatory interleukins (e.g. IL-1β and TNF-α), and chemokines (e.g.MCP-1 and MIP-1α). Resident inflammatory cells like microglia can also be activated and proliferated, producing pro-inflammatory factors (Jin et al. 2013). Astrocytes also contribute to cell damage and death following cerebral ischemia by secreting major histocompatibility complex and costimulatory molecules (Jin et al. 2013).

By activating platelet-activating factor in mice with ischemic strokes, GB switched microglia/macrophage polarization from the inflammatory M1 phenotype to the anti-inflammatory M2 phenotype in vivo and in vitro (Shu et al. 2016). GB attenuated the activation of microglia and decreased the production of IL-1β and IL-18 in rat brains with hypoxic stress (Chen et al. 2018b). NLRP3 (NLR family, pyrin domain containing 3) inflammasome provided an important molecular mechanism in the induction of the central pro-inflammatory cytokine IL-1β via activation of caspase-1 (Haneklaus and O'neill 2015). In neonatal hypoxic-ischemic brain injury model of rat pups, GB reduced NLRP3 expression, mainly in microglia, and significantly inhibited the expression of Caspase-1 and the nuclear translocation of NF-κB P65, preventing NLRP3 inflammasome activation (Chen et al. 2018c). An anti-inflammatory effect of GB derivative XQ-1H has also been reported. XQ-1H suppressed neutrophils infiltration and inflammatory mediators, including cerebral ischemia induced intercellular adhesion molecule 1 (ICAM-1) and matrix metalloproteinase-9 (MMP-9), into the ischemic region of the brain. Downregulated MMP-9 expression could reduce extracellular matrix degradation, and protect BBB via tight junction, which could alleviate BBB compromise caused influx of immunocytes and edema formation (Wei et al. 2013). Furthermore, in SD rats with MCAO, pretreatment with bilobalide improved neurological function and increased SOD activity while it decreased infarct volume, brain edema, and the concentrations of MDA, nitric oxide, TNF-α, IL-1β (Jiang et al. 2014). Bilobalide also activated JNK1/2 and p38 MAPK but not ERK1/2 (Jiang et al. 2014). In line with the in vivo results, pretreatment with bilobalide reduced nitric oxide, TNF-α, IL-1β, p-JNK1/2, and p-p38 MAPK expression in the cortical neurons of rats after oxygen-glucose deprivation and reoxygenation injury (Jiang et al. 2014).

### Neuron death and apoptosis

Neuron apoptosis plays a large role in cell death after ischemic strokes. A previous review identified that there are two general pathways by which apoptosis is triggered by ischemic strokes: the first follows cytochrome c release from mitochondria and consequential caspase 3 activation; the other centers around the activation of death receptors that are expressed on the membrane and caspase 8 activation (Broughton et al. 2009). Various factors have been shown to induce neuron apoptosis, such as disruption of calcium homeostasis, oxidative stress, DNA damage, and neuroinflammation (Broughton et al. 2009; Shin et al. 2018). Another factor was the increased expression of purinergic 2X7 receptor (P2X7R) and NLRP3 inflammasome in ischemic brain tissue after stroke, the inhibition of which by their inhibitors inactivated caspase 3 and reduced neuronal apoptosis (Ye et al. 2017).

In vitro, GB and ischemic preconditioning improved cell viability and inhibited neuronal apoptosis by stimulating astrocytes to express and secrete erythropoietin (Wu et al. 2013). Exposing cortical neurons to ischemic conditions caused the release of lactate dehydrogenase (LDH) and the upregulation of a stress-related protein RTP801 (Wu et al. 2015). GB reduced LDH release and RTP801 induction, resulting in an improvement in cell survival (Wu et al. 2015). GA and GB restrained neuronal apoptosis in a SD rat model of permanent focal cerebral ischemia (pMCAO) where GA and GB were dissolved in saline and injected intravenously after pMCAO. Production of ROS, activation of mitochondrial pro-apoptotic molecules, including cytochrome c, caspases 3 and 9, and PARP, and phosphorylation of c-Jun N-terminal kinase (JNK) in ischemic penumbra were all blocked by GA and GB treatment (Wang et al. 2014). Bilobalide treatment suppressed caspase 3 cleavage, reduced the LC3-II/LC3-I ratio, and promoted angiogenesis in rat brains following MCAO by activating the NOS and Akt pathways (Zheng et al. 2018b). In vitro, the protective effects of bilobalide on neurons have been examined under Aβ1–42, H_2_O_2_, and serum deprivation conditions. Bilobalide enhanced cell viabilities, inhibited apoptosis, attenuated mitochondrial membrane potential depolarization, and suppressed caspase 3 cleavage through the PI3K/Akt signaling pathway (Shi et al. 2010).

### Cognitive impairment

In the acute phase of ischemic strokes, declines in cognitive efficiency in domains including executive function, immediate recall, delayed recall, speech, divergent thinking, attention and concentration, and visual-constructive performance have been observed (Bugarski Ignjatovic et al. 2015). Body mass index, hypertension, low optimism, and physical function have also been associated with a decrease in global cognition (Vaughan et al. 2016). Furthermore, serum oxLDL levels and enlarged perivascular spaces were also associated with cognitive impairment in patients that suffered ischemic strokes (Wang et al. 2018b, Arba et al. 2018). Some studies have observed beneficial effects of *G. biloba* on cognitive impairment in neurodegenerative diseases and ischemic strokes (Yuan et al. 2017; Li et al. 2017). EGB1212 is another standard extract of *G. biloba*. Pre-treatment and post-treatment with EGB1212 increased both hippocampal neuron survival and spatial learning and memory functions after global cerebral ischemia/reperfusion in rats (Yin et al. 2013). However, Vaghef and Bafandeh Gharamaleki (2017) reported that pretreatment with *G. biloba* was not as effective as exercise in recovering from memory impairment, although its effect on oxidative stress in the hippocampus was supported. Moreover, GK alleviated neurological impairments and accelerated angiogenesis of the ipsilateral cortex and striatum injured by tMCAO in a mouse model (Chen et al. 2018c). These effects were attributed to the activation of the JAK2/STAT3 pathway and upregulation of HIF-1α and VEGF (Chen et al. 2018c). A protective effect of bilobalide on cognitive impairment has also been demonstrated in mice. Wu et al. (2016) found that chronic administration of bilobalide intraperitoneally improved depression-like behavior and cognitive deficiencies induced by chronic unpredictable mild stress in mice.

## Conclusion and perspectives

The neuroprotective effects of ginkgolides and bilobalide have been validated in multiple preclinical studies. In recent decades, the advancements in animal models and imaging techniques help us witness the appreciation of the significance of the ginkgolides and bilobalide in the cellular and signaling events of cerebral ischemia, including excitotoxicity mechanisms, inflammatory pathways, oxidative damage, metabolic disturbance, apoptosis and neuroprotection. As a leading cause of mortality and mobility in modern society, stroke affects people’s life both in developed and developing countries. However, limited treatment options currently exist, only for a small proportion of stroke victims, making it vital to develop effective treatments. Now, researchers are also focusing on the design and synthesis of ginkgolides and bilobalide analogs with brain-targeting ability, which would cause effective and continuous therapy for central nervous system diseases in the future.

## Data Availability

Not applicable for this review article.

## Abbreviations

BBB: Blood brain barrier
GA: Ginkgolide A
GB: Ginkgolide B
GC: Ginkgolide C
GJ: Ginkgolide J
GK: Ginkgolide K
GL: Ginkgolide L
GM: Ginkgolide M
I/R: Ischemia/reperfusion
MCAO: Middle cerebral artery occlusion
OGD/R: Oxygen and glucose deprivation, reperfusion or reoxygenation
rtPA: Plasminogen activator
